# A castor oil plant (*Ricinus communis*)-derived implant improves the biomechanical properties of noncritical bone defects

**DOI:** 10.1590/ACB360202

**Published:** 2021-02-22

**Authors:** Cecilia Hernández-Flores, Alfonso Delgado, Victor Manuel Domínguez-Hernández, Rene Valdez-Mijares, Victor Manuel Araujo-Monsalvo

**Affiliations:** 1 MSc. National Rehabilitation Institute LGII – Biochemistry Service – Ciudad de México, Mexico.; 2 MD, D.Sc. Autonomous University – Chihuahua School of Medicine – Department of Physiology – Chihuahua, Mexico; 3 PhD. National Rehabilitation Institute LGII – Biochemistry Service – Ciudad de México, Mexico.; 4 MSc. National Rehabilitation Institute LGII – Biochemistry Service – Ciudad de México, Mexico.; 5 PhD. National Rehabilitation Institute LGII – Biochemistry Service – Ciudad de México, Mexico.

**Keywords:** Ricinus, Tibia, Polyurethanes, Rats

## Abstract

**Purpose:**

The biomechanical properties of the polyurethanes implant material derived from castor oil plant (Ricinus communis) were evaluated in a noncritical bone defect model in rat tibia.

**Methods:**

After three weeks of the implant application, the tibias were tested by means of the biomechanical three-point flexion test and resistance, rigidity, energy at maximum load and maximum energy were evaluated. Nonparametric statistical analysis was performed.

**Results:**

It was found that the group that received the implant behaved the same as the intact control group and also showed a significant increase in maximum load compared to the spontaneous repair group.

**Conclusions:**

Our results indicate that the tibias with the implant material in a noncritical bone defect recover normal biomechanical parameters in less time than spontaneously.

## Introduction

Loss of bone segments causes human and animal health problems. The most frequent causes of loss of bone segments are resection of tumors, bone infections and trauma. The use of existing materials, the development and use of new materials to optimize, accelerate, promote and facilitate the repair of bone defects, both those that repair themselves spontaneously (noncritical), and those that require medical intervention for their repair (critical) is of high importance for orthopedic surgery, dental surgery and maxillofacial surgery^1^^–^ 3.

Bone defects had been treated with biological allografts or synthetic grafts. Numerous bioactive bone substitutes such as hydroxyapatite, coral-collagen composites, bioactive glass, natural coral and calcium phosphate cements had been studied^4^.

Implant material castor oil plant (*Ricinus communis*)-based polyurethanes from two components, polyol and prepolymer, obtained by modification of the castor oil plant, has been employed as a biomaterial as a space filler, minimizing the local production of fibrous tissue^5^. In culture, the implant together with mesenchymal stem cells revealed that it does not affect cell adhesion or proliferation and increases the formation of mineralization nodules^6^; therefore, it was decided to evaluate its effect on repair of a noncritical bone defect in rat tibia through biomechanical analysis.

## Methods

All animal procedures were performed according to the National Rehabilitation Institute Guide for Care and Use of Laboratory Animals, compliant with the National Institutes of Health (NIH, USA) Guide for Care and Use of Laboratory Animals.

Twenty-one male Wistar rats weighing 300 ± 40 g body weight were housed with a light-to-dark cycle of 12:12 and fed and watered on demand. Three groups of seven rats each were randomly selected and organized in the following manner: G-1, untreated age- and weight-matched control rats; G-2, rats with an unfilled right tibia defect, rats were allowed to recover for three weeks to address bone defect spontaneous repair; G-3, rats with a right tibia defect filled with the test implants, rats were left to recover for three weeks.

### Bone defect

A noncritical bone defect was practiced in the right tibia (RT) of each animal. Anesthesia was induced with intraperitoneal sodium pentobarbital (50 mg/kg, i.p.), the experimental member was shaved and washed with iodopovidone (8 g/100 mL; Dermodine, DEGASA). A 1-cm incision was made on the tibial crest, taking care not to damage the underlying bone or the adjacent muscle. The superficial fascia was separated from the skin and the tibia was exposed. A 1-mm diameter unicortical defect was made in the region of interest using an electric drill (Mini drill Pros Kit Model PK-500) with a ball-shaped tungsten burr for bone surgery and the implant was placed into it; finally, the wound was closed (000 Atramat surgical silk, México). All animals were monitored every third day, verifying their general health status. The body weight of each animal prior to sacrifice was recorded in a CO_2_ chamber.

### Implant material

Implants were prepared as directed from the BioOsteo kit (Biomecânica, São Paulo, Brazil) using a mixture of standard proportions (1:1:0.85) of prepolymer, calcium carbonate and polyol (polyurethanes derived from castor oil plant *Ricinus communis*).

### Biomechanical test

**Figure 1:**
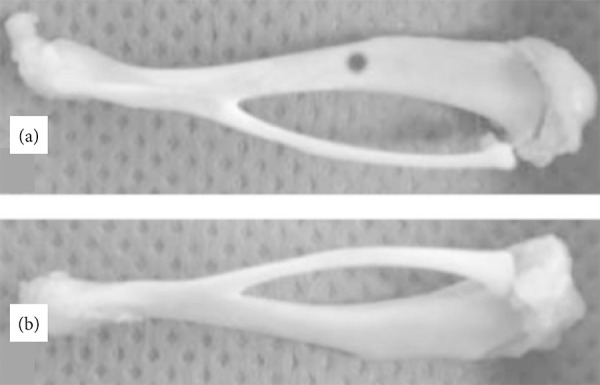
Bone defect. (**a**) Tibia defect is performed with an electrical surgical drill through only one cortex at the location shown at middle of the tibia; (**b**) The intact, control side (*left*) is added for comparison.

**Figure 2:**
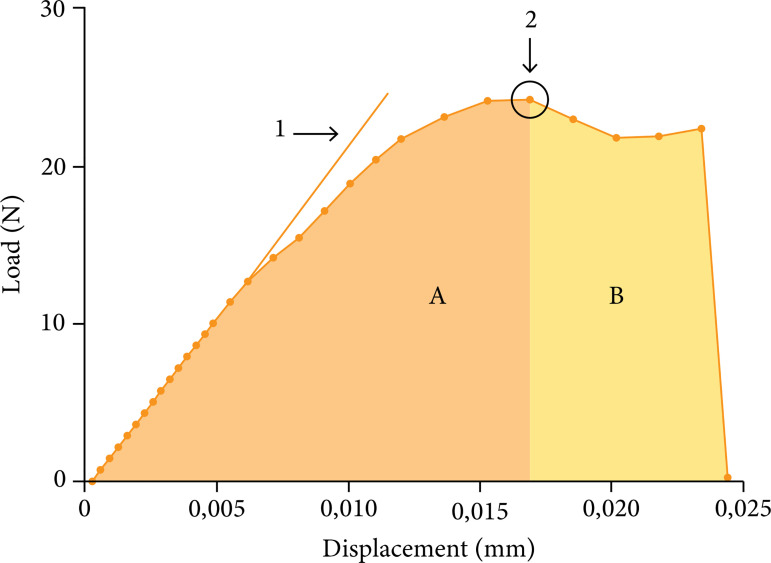
Obtaining the biomechanical parameters. 1) Rigidity: slope of the load-displacement curve in its initial linear portion. 2) Resistance: maximum load recorded, highest point of the graph. 3) Energy at maximum load: area B under the curve, dark gray. Energy so that the tibia reaches its point of greatest resistance. 4) Maximum energy: total area under the curve (area A + area B). Total energy to failure.

The tibias were dissected and their length was measured. Destructive biomechanical three-point bending tests were performed on a universal testing machine (Instron 4502, Instron Inc., Canton, MA) with a 1-kN load cell. The right tibia (RT) (Fig. 1a) was placed between two round bars separated at a distance of 14 mm on the traction side, taking care that the tibia is aligned on the bars of the device to the center of the supports, and a preload of 3.6 ± 0.1 N was applied on the opposite side. The tests were performed at a speed of 2.5 mm/min until fracture. To the left tibia (LT) (Fig. 1b) the load was applied to the same level of the RT following the same procedure. All tests were performed in the first 30 min after sacrifice. The displacement load curves were recorded and captured on a conventional computer^4^^–^ 7. Stiffness, resistance, energy at maximum load and maximum energy (Fig. 2) were calculated from each graph obtained through the Origin 8 program (OriginLab, MA, USA). For each group, the measurements were normalized with the LT of each animal.

### Statistical analysis

Based on the sample size and assuming non-normality of the data, statistical analysis was performed with the nonparametric Mann–Whitney U test to compare between groups^8^. The level of significance was p < 0.05 and the analysis was performed with the software SPSS 9.0 (SPSS Inc., Chicago IL, USA).

## Results

**Figure 3:**
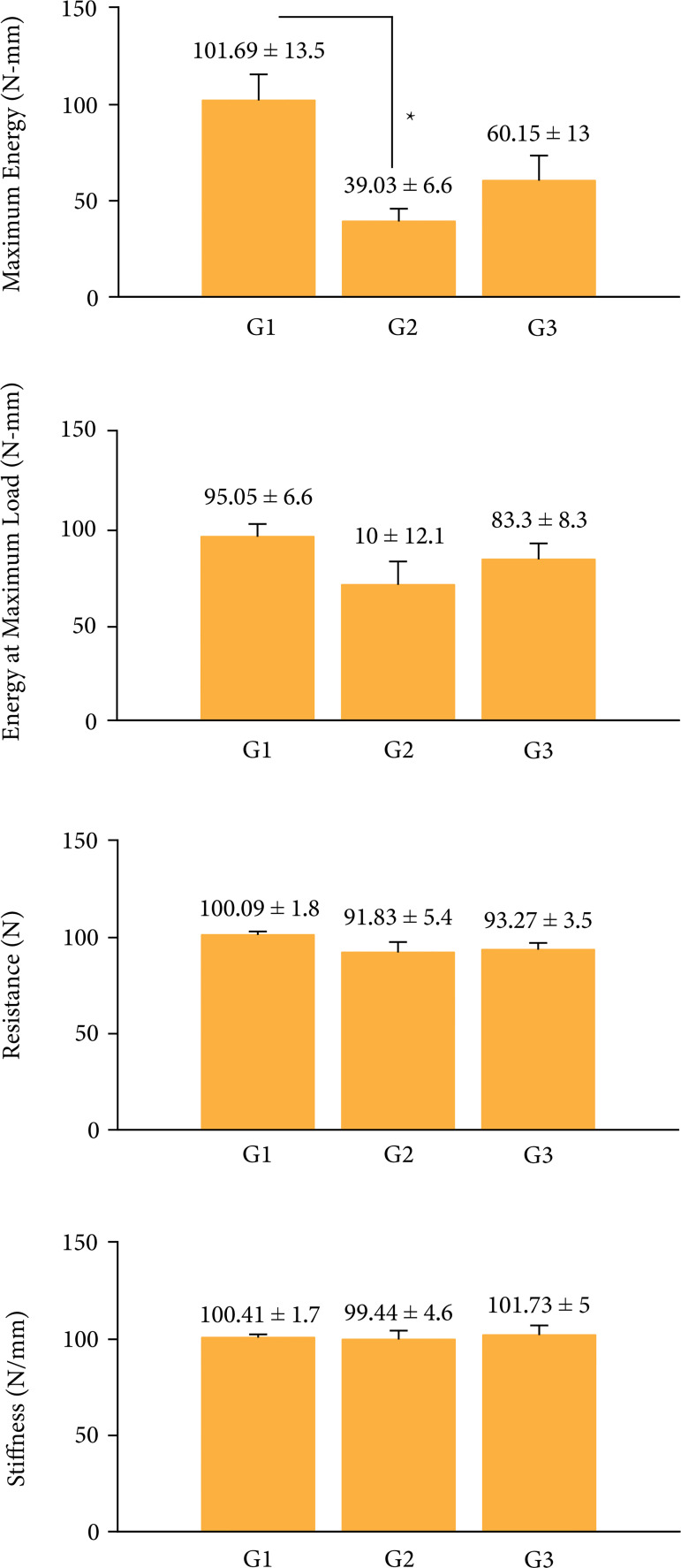
Biomechanical parameters analyzed between groups (figures are mean ± standard error of the mean); *p < 0.5.

The rats were in general good health, the experimental limb was bearing rat’s weight without limping and showed an increase in body weight according to their age and strain. Body weight at sacrifice was 400 ± 31.8 g on average. From the biomechanical trial, 71.4% of the fractures of the three groups were transverse at the site of the defect and 28.5% were short obliques. The average length for the tibias was 42.2 ± 1.1 mm. The results of the U Mann–Whitney test (Table 1 and Fig. 3) between G-1 vs. G-3 did not show significant differences for any of the measured parameters. Groups G-1 vs. G-2 revealed differences for maximum energy.

**Table 1:** Results of the Mann–Whitney U test.

		Maximum Energy (N-mm)		Energy at Maximum Load (N-mm)		Resistance (N)		Stiffness (N/mm)
G1 vs G2		0.001*		0.097		0.097		1.000
G1 vs G3		0.073		0.535		0.053		0.805

* Statistically significant difference p < 0.05.

## Discussion

In this work we evaluated a noncritical bone defect on the anteromedial surface of the rat tibia to which the implant was placed for 3 weeks. This time window of analysis was chosen because it has been demonstrated^9^ through histopathological, histochemical and morphometric studies that, after 3 weeks, a noncritical bone defect is filled with new bone, but not repaired. In addition, it has been reported^1^ that at 21 days a noncritical bone defect in the rat skull has not yet been repaired. Furthermore, there is scanning electron microscopy evidence^10^ that within 14 days a fracture in the rat tibia is not yet repaired, the bone callus is observed but is subsequently calcified at 30 days.

Accordingly, for G-1 vs. G-2 groups, a significant difference was found only for maximum energy (Fig. 3, Maximum Energy), this indicates that the defect, 3 weeks after surgery, is still in the process of repair and therefore still does not have the biomechanical properties of a healthy tibia, this result agrees with the selection of testing time frame (see above); furthermore, a study in rat tibia with a critical bone defect treated for 8 weeks reports that they only found significant differences for energy, without complete repair of the defect^11^.

For groups G-1 vs. G-3, no significant difference in any of the biomechanical parameters analyzed was found, this suggests that group G-3 shows the biomechanical properties of a healthy tibia in less time than spontaneous repair. Laureano *et al*.^12^ reported that four weeks in a rabbit model with 2 bone defects in the calvaria, during the initial evaluation period it was possible to identify the presence of particles of this implant surrounded by fibrous connective tissue, that is, a short time for complete bone repair; after 15 weeks they observed an almost complete bone repair, the implant had a positive influence on bone neoformation in the defect. In this work, the repair time was reduced to 3 weeks. The type of implant used here has been studied through histology and it has been reported that in bone defects of the rat jaw at different times they observe that it is biocompatible and osteintegrable^13^; furthermore, Nacer *et al*.^14^, in a 2-mm defect in the rat femur, found that after 15 days of repair the group with the implant showed newly formed bone tissue defect at the margins of the bone with osteogenic activity inside the implanted material; at 30 days they found the presence of osteocytes trapped in the hollows of the bone trabeculae, which indicates the maturing of newly formed bone tissue; and after 60 days, a large area containing mature bone tissue was observed along with a greater amount of osteocytes in the margins and inside the defect, the osteoblastic activity was maintained and there was a large concentration of mature osteocytes^14^. Moreover, it has been reported that the implant has bone neoformation due to osteoconduction, partial resorption or very little resorption^15^^,^ 16. All these evidences give support to these findings and indicate that the implant was tested on nonrepaired bone.

Taken together, our results show that the implant favors an early recovery of biomechanical properties in a noncritical bone defect. In previous works, some authors report contradictory effects regarding the resorption of the implant; however, from the biomechanical point of view, it helps to recover the biomechanical properties similar to a normal tibia; in addition, the implant can be used to fill bone defects^5^^,^ 16, because bone cells adhere, proliferate and promote differentiation in the presence of this kind of implant^6^; new bone formation has been observed as well with a low inflammatory process and low production of fibrous tissue^5^. Macroscopic studies by means of biomechanical analysis can be complemented by radiographic, histomorphometric and immunohistochemical studies in order to study the mechanisms of the implant in the bone at the microscopic level.

## Conclusions

Our results indicate that the tibias with the polyurethanes derived from castor oil plant (*R. communis*) in a noncritical bone defect recover the biomechanical parameters more quickly than if they are left to spontaneously repair, consequently they can contribute to reduce patient’s hospital stay and therapy costs.
